# Determinants of Physiological Resilience Among Recreational‐To‐Competitive Endurance Runners

**DOI:** 10.1111/sms.70349

**Published:** 2026-07-23

**Authors:** Timi Malinen, Pekka Matomäki, Arja Uusitalo, Heikki Kyröläinen, Olli‐Pekka Nuuttila

**Affiliations:** ^1^ Faculty of Sports and Health Sciences University of Jyväskylä Jyväskylä Finland; ^2^ Paavo Nurmi Centre & Unit for Health and Physical Activity University of Turku Turku Finland; ^3^ Department of Sports and Exercise Medicine, Clinicum University of Helsinki Helsinki Finland; ^4^ Helsinki Clinic for Sports and Exercise Medicine, Foundation for Sports and Exercise Medicine Helsinki Finland; ^5^ UKK Institute for Health Promotion Research Tampere Finland

**Keywords:** durability, endurance performance, endurance running, fatigue

## Abstract

The aim of this study was to investigate (1) whether factors related to endurance performance are associated with physiological resilience in recreational‐to‐competitive runners, and (2) differences between recreational and competitive runners in physiological resilience. During three test days, 33 runners (15 competitive and 18 recreational) were tested for their peak fat oxidation, maximal blood lactate concentration (La_max_) after maximal anaerobic running test (MART), maximal voluntary contraction (MVC) in isometric leg press, maximal oxygen uptake (VO_2max_), maximal speed of incremental test (sPeak), speeds at ventilatory thresholds (sVTs), running economy (RE), and physiological resilience. Physiological resilience was tested as deterioration of VO_2max_, sPeak, VTs, and RE during and after ~2.5 h moderate‐intensity run at ~90% of VT1. Associations between other variables and physiological resilience were analyzed using Pearson's or sex‐adjusted partial correlations in the whole sample. Recreational runners had higher deterioration of VT1 (*p* = 0.049), but lower deterioration in RE (*p* = 0.042) than competitive runners. Lower decline in sPeak was associated with higher VO_2max_ (*r* = 0.37, *p* = 0.04) and lower La_max_ (*r* = −0.37, *p* = 0.03). Lower decline in sVT1 was associated with higher VO_2max_ (*r* = 0.37, *p* = 0.04), sVT2 (*r* = 0.52, *p* = 0.002), sVT1 (*r* = 0.48, *p* = 0.006), and sPeak (*r* = 0.44, *p* = 0.01). Lower decline in RE was associated with worse RE (*r* = −0.49, *p* = 0.004) and higher MVC (*r* = −0.40, *p* = 0.02). Lower decline VO_2max_ was associated with worse RE (*r* = 0.42, *p* = 0.02). It appears that physiological resilience is associated with multiple different physiological systems. High aerobic fitness and MVC of the leg extensors, and lower La_max_ after MART may be related to better physiological resilience.

## 1 Introduction

Endurance performance has usually been determined by the Joyner model, which includes maximal oxygen consumption (VO_2max_), first and second lactate or ventilatory thresholds (LTs and VTs), and movement economy (e.g., running economy, RE) [1]. In addition, anaerobic capacity and neuromuscular system performance have shown to affect distance running performance [2]. Physiological determinants of endurance performance are not stable, but they rather deteriorate during exercise or competition. The ability to resist the deterioration is highly individual and has, in recent years, been called “physiological resilience” [3] or “durability” [4]. Physiological resilience has been shown to be a crucial variable for an athlete competing in endurance sports (e.g., distance running). For example, inclusion of physiological resilience in the prediction equations for marathon times has improved the ability to predict the finishing times of athletes [5] and marathon finish times has been shown to be associated with the ability to prevent decline in LT2 [6].

Physiological resilience has been studied with different variables in the past, including decline in VTs [7], LTs [8], VO_2max_ [9, 10], RE [11], and critical power [12]. Currently the determinants of the ability to resist the decline in these variables are not well‐known. It has been proposed that they generally overlap with the physiological factors associated with endurance performance (e.g., higher proportion of type I muscle fibers, greater mitochondrial volume, oxidative capacity, and capillary density) [3, 5]. Decline in a certain variable indicates that physiological determinants of the variable have deteriorated. As the variables in the Joyner model are determined by different physiological systems (VO_2max_ by the maximal oxygen transport capacity [13], RE by mechanical and metabolic efficiency [14] and speeds at the VTs by oxidative capacity and RE [7, 15]), the decline in them is caused by perturbation of the distinct physiological systems. Therefore, the resilience of these different variables is possibly determined by different factors. Understanding the determinants of physiological resilience would help in designing training programs that focus on specific factors to enhance resilience of endurance athletes.

Research regarding determinants of physiological resilience has so far resulted in contradicting findings. Gallo et al. [16] showed that the decline in VT1 during moderate‐intensity cycling is highly individual regardless of the non‐fatigued levels of VO_2max_, VTs, gross efficiency, or peak fat oxidation. Similar results have been shown in other respective studies [17]. In contrast, a study by Spragg et al. [18] showed that the decrement of critical power after severe intensity cycling was associated with multiple laboratory‐derived physiological variables and substrate utilization at submaximal workloads. Differences between the studies might arise from differences in the fatiguing protocol and participant populations, but it still raises the question of whether physiological resilience could be explained by individual differences in traditional determinants of endurance performance.

In addition to the traditional determinants of endurance performance, anaerobic and neuromuscular capabilities [2] and fat utilization capacity [19] affect endurance performance and have been proposed to be related to physiological resilience [3, 5]. Strength training interventions have resulted in improved physiological resilience, and thus maximal strength level might be related to resilience [20, 21, 22, 23]. Individuals with higher maximal strength use relatively lower force during running, which might improve physiological resilience by limiting the need to recruit more muscle fibers [24]. In contrast, greater anaerobic capacity is associated with a higher proportion of fast type II muscle fibers [25, 26] which further has been associated with greater fatigability and slower recovery [27, 28]. A higher proportion of type I muscle fibers might limit the need to recruit less‐efficient type II muscle fibers [29] and therefore improve physiological resilience.

Depletion of glycogen stores is the limiting factor of moderate‐intensity exercise and plays a part in fatigue in heavy‐intensity exercise as well [30]. Higher capacity to oxidize fat, at submaximal intensities, saves muscle and liver glycogen, which is essential for performance [31]. In addition, reduced fat oxidation accelerates glycogen use compared to higher fat oxidation [32]. In applied settings, studies have shown that fat oxidation rate at the beginning of exercise is associated positively with time to task failure at a moderate intensity and the beginning of the decline in VT1 [16], and that carbohydrate ingestion during prolonged exercise blunts the decrement of physiological profile compared to placebo [33, 34, 35]. These findings imply that fat oxidation capacity might play a role in determining physiological resilience.

The variables in the Joyner model differentiate athletes of different fitness levels [36] but it is currently less clear how the deterioration of the variables differs between fitness levels. It has been shown that the resilience of running economy [9, 11] and VO_2max_ [9] decrease more in the less trained than in highly trained individuals but no comparisons between other variables have been reported.

The purpose of this study was to investigate the associations between the non‐fatigued physiological profile (Joyner model, fat oxidation capacity, anaerobic capacity, and neuromuscular performance) and physiological resilience (as measured by decline in VO_2max_, sPeak, VTs, and RE after ~2.5 h of moderate intensity running). In addition, this study compared the differences in physiological resilience between competitive and recreational runners.

## 2 Materials and Methods

### 2.1 Participants

Based on previous studies regarding physiological resilience in endurance running, 10–15 participants per group are needed for significant decline in thresholds, RE or VO_2max_ [8, 9]. Therefore, a total of 45 well‐trained endurance runners voluntarily participated in the current study and gave their written consent. Of the participants, 20 were competitive (10 females) and 25 were recreational runners (13 females). The caliber of the participants was evaluated by McKay et al. [37], and competitive runners were identified as tier 3 or higher and recreational runners as tier 2. Eligibility criteria were (i) to be healthy and free of musculoskeletal injuries, (ii) running ≥ 3 times per week, (iii) to have raced a half‐marathon or race of equivalent duration in the past 12 months, and (iv) for competitive runners to race in the elite category of their sport at the national and/or international level, and for recreational runners to have completed a half‐marathon in less than 2 h or a similar result in triathlon, ultrarunning, or orienteering. The study was approved by the Ethics committee of University of Jyväskylä (1581/13.00.04.00/2024) and conducted in accordance with the Declaration of Helsinki. Prior to the first laboratory visit, participants filled in questionnaires regarding their health status and training history to verify their eligibility to participate in the study and went through resting electrocardiography verified by a licensed physician.

### 2.2 Study Design

**FIGURE 1:**
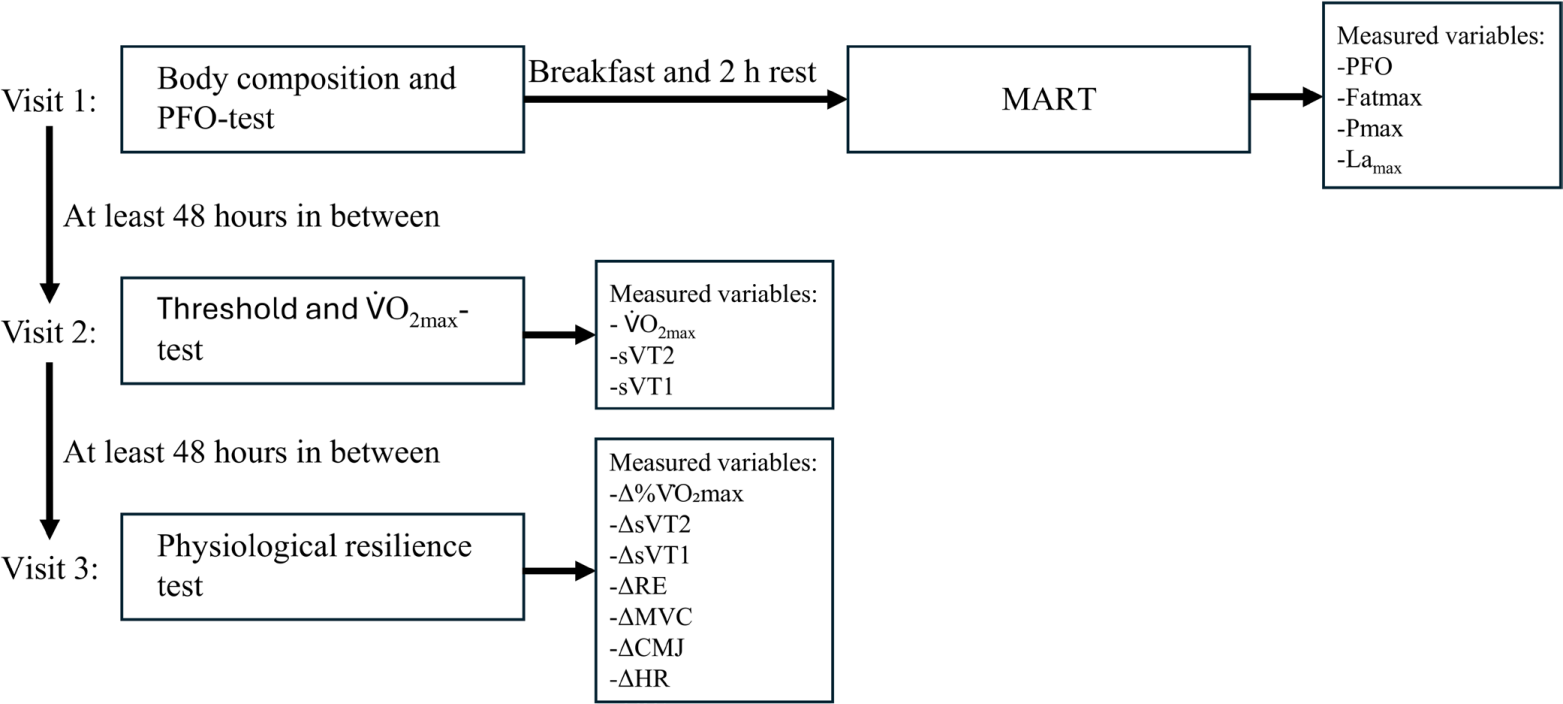
Study design, measurements, and measured variables. CMJ, counter movement jump; Fatmax, intensity of peak fat oxidation relative to maximal oxygen uptake; HR, heart rate; La_max_, maximal lactate concentration from MART; MART, maximal anaerobic running test; MVC, maximal voluntary contraction; PFO, peak fat oxidation; P_max_, maximal power from MART; RE, running economy; sVT1, speed at ventilatory threshold 1; sVT2, speed at ventilatory threshold 2; V̇O_2max_, maximal oxygen uptake.

The study was conducted in the facilities of University of Jyväskylä, and all measurements were done with the same laboratory setting. All participants visited the laboratory on three different occasions (Figure 1). During the first visit, participants went through a peak fat oxidation test, maximal anaerobic running test (MART), and a body composition measurement with InBody 970 device (Biospace Co. Ltd., Seoul, Korea). The peak fat oxidation test and body composition assessments were completed in a fasted state. After eating a small free‐choice breakfast and resting for at least 2 h, participants performed a maximal anaerobic running test (MART). During the second visit, they performed threshold‐ and VO_2max_‐tests to determine non‐fatigued VO_2max_, maximal speed in VO_2max_‐test and VTs. In the third visit, they performed a ~2.5 h physiological resilience test to measure the changes in the VO_2max_, maximal speed in the VO_2max_‐test, VTs and RE during prolonged moderate intensity running at ~90% of VT1. During running test visits, they performed countermovement jumps and isometric leg press tests. The visits were separated by a minimum of 48 h and a maximum of 10 days. The participants were instructed to wear similar clothing in all tests, use the same footwear in the running tests, refrain from caffeine for 12 h, and refrain from alcohol and intense exercise for 24 h before each measurement.

### 2.3 Measurements

#### 2.3.1 Peak Fat Oxidation Test (PFO)

The peak fat oxidation test was a modified version of Amaro‐Gahete et al. [38]. The participants arrived at the laboratory in the morning (7:00–9:00 a.m.) after 10–12‐h fast. After arrival, height and body mass, measured in running clothes, were taken to the closest 0.1 cm and 0.1 kg, respectively. The test consisted of 3‐min walking stages. The starting speed of the test was 5 km/h and gradient 0°, after which the speed was increased to 6 km/h. After the second stage, the speed was kept at 6 km/h and the gradient was increased by 1.2° every stage. During the test, ventilatory gases were monitored continuously on a breath‐by‐breath basis (Jaeger VyntusTM CPX, CareFusion Germany 234 GmbH, Hoechberg, Germany). The test continued until the participants' RER increased to 0.95 [38].

#### 2.3.2 Maximal Anaerobic Running Test (MART), Countermovement Jump (CMJ), and Isometric Leg Press

MART was completed during the same day as the PFO‐test, after at least 2 h of rest. The test was done according to Nummela et al. [39], and it started with a standardized warm‐up of 5 min at 8.1 km/h and consisted of running bouts of 20 s with 100 s of recovery between each bout. The starting speed was 11.3 km/h for females and 12.7 km/h for males. The speed was increased by 1.4 km/h every stage, and the inclination of the treadmill was kept at 3° during the test. When 40 s of the rest had passed, a blood sample was obtained from the fingertip to analyze the BLa with a Biosen C‐line lactate analyzer (EKF Diagnostic, Magdeburg, Germany). The treadmill started 5 s before the rest was completed to account for the acceleration of the treadmill. The test continued until volitional exhaustion. Immediately after exhaustion, BLa was taken at 1, 3, and 5 min to determine the peak BLa concentration. Before and after the test, participants performed countermovement jumps (CMJ) and isometric leg press tests to familiarize themselves with the movements. CMJs were performed on a contact mat, and participants were instructed to descend to a self‐selected depth, jump maximally upwards with hands on hips, and land with straight legs on the midfoot. Isometric leg press tests were performed at a knee angle of 108° using a custom‐made isometric leg press. Following warm‐ups with 50%, 75%, and 90% of their self‐estimated maximal force, they executed three maximal contractions with 30 s of rest between trials.

#### 2.3.3 Threshold Test and VO_2max_ ‐Test

During the second visit, a threshold test for assessing VT1 and a VO_2max_‐test to assess V̇O_2max_ and VT2 was completed. The incremental test consisted of 3 min‐stages with speed increasing by 1 km/h every stage. Starting speed was 7–10 km/h depending on the previous self‐reported race results of the participant. Inclination of the treadmill was kept at 1% [40]. The treadmill was stopped after every stage to collect a fingertip blood sample to assess BLa concentration (Biosen C‐line lactate analyzer, EKF Diagnostic, Magdeburg, Germany). The test continued until RER reached and stayed continuously over 1.00 during the last minute of the stage. After the incremental test, participants rested for 10 min after which the VO_2max_‐test started. The starting speed for the V̇O_2max_‐test was 2 km/h higher than the starting speed of the incremental test. The speed increased by 1 km/h every minute until volitional exhaustion. During both tests HR (Polar H10, Polar Oy, Kempele, Finland) and breath‐by‐breath ventilatory gases (Jaeger VyntusTM CPX, CareFusion Germany 234 GmbH, Hoechberg, Germany) were monitored continuously.

#### 2.3.4 Physiological Resilience Test

During the third visit, participants completed a physiological resilience test that has been previously reported on [41]. When the participants arrived at the laboratory, their weight was measured, with running clothes and shoes on, and then they completed the same warm‐up as during the second visit. After the warm‐up, they executed countermovement jumps on a contact mat following the same protocol as during the MART. After countermovement jumps, the isometric leg press test was performed using the same custom‐made isometric leg press and protocol as in prior visits. Participants completed three maximal contractions.

**FIGURE 2:**
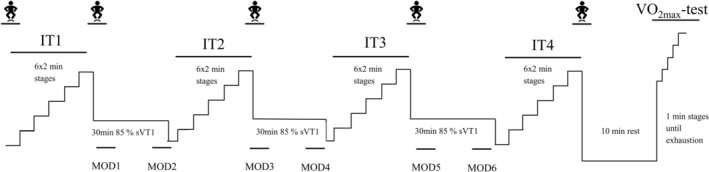
The physiological resilience test protocol. IT: Incremental test consisting of 6 2‐min stages to measure first ventilatory threshold (VT1); MOD1‐6: Timepoint when ventilatory gases were monitored during moderate intensity running at 85% of the speed at VT1 (sVT1) to measure running economy (RE); V̇O_2max_‐test: Maximal oxygen uptake (VO_2max_) test to measure VO_2max_, maximal speed of the test (sPeak), and second ventilatory threshold (VT2).

The physiological resilience test protocol (Figure 2) was adapted from prior studies using moderate‐intensity running [7, 8, 17], and incremental tests during the process to assess VT1 [16]. It consisted of four short incremental tests (IT1‐4) to determine VT1 and the time‐course of its change, and three 30‐min running bouts. Each IT included six 2‐min stages beginning at a speed 2.4 km/h below the sVT1 measured during visit 1 and speed increasing by 0.8 km/h. During each of the ITs, the treadmill ran continuously. Breath‐by‐breath respiratory gases were continuously monitored during ITs. After the final stage, participants stood for 60 s for BLa sampling, after which the mask was removed, and three maximal CMJs were performed. Participants were then re‐weighted with shoes on.

Between ITs, participants completed 30 min of moderate intensity running (MOD) at 85% of sVT1 from visit 1. 85% of VT1 was chosen to average the speed of the test to about 90% of sVT1 with the ITs. Breath‐by‐breath respiratory gases were recorded throughout the first and last 5 min of each MOD, from which RE was analyzed. The results in the different timepoints will be referred to as MOD1–MOD6 in the following sections (14–19 min [MOD1], 39–44 min [MOD2], 59–64 min [MOD3], 84–89 min [MOD4], 104–109 min [MOD5], and 129–134 min [MOD6] from the start). The three MODs and four ITs lasted 138 min in total. Participants removed the mask while running but the treadmill was stopped for ~15 s, 5.5 min before the end of each MOD to put the mask back on. Following the MOD, participants stood still for 1.25 min for BLa sampling, after which the next IT started. Participants were permitted to drink water ad libitum when not wearing the mask but were not allowed to consume exogenous energy. Participants were permitted to use the toilet during the MODs, but the stoppage was timed, and full minutes were added to the MOD to ensure 30 min of running. An electric fan was placed approximately 2 m in front of the participants, directed at the torso and lower body, and operated at a constant speed throughout.

After completing the four ITs and three MODs, participants rested for 10 min after which they performed the same VO_2max_‐test as during the first visit, followed by the isometric leg press test. At the midpoint of the 10‐min recovery period, participants rinsed their mouths with 200 mL of a 6% carbohydrate solution. They were instructed to repeat the following: take a mouthful, rinse for 5 s, and expectorate, until the solution was rinsed. Participants were advised not to swallow the solution. The purpose of the rinse was to enhance motivation during the VO_2max_‐test and minimize the influence of central fatigue, without ingesting carbohydrates, as the study aimed to measure maximal physiological capacity. Carbohydrate mouth rinsing has been shown to activate brain regions associated with motivation and improve endurance performance [42], though it has not been seen to improve maximal performance during 15‐min maximal exercise [43] or incremental exercise test with 1‐min stages [44].

### 2.4 Measures

#### 2.4.1 Peak Fat Oxidation and Fatmax

Peak fat oxidation (PFO) and the VO_2_ of which PFO occurred (fatmax) were calculated from the VO_2_ and VCO_2_ collected during the last 1 min of each stage. Fat oxidation was calculated by using Equation (1) [45]. The PFO was determined by plotting the fat oxidation rate and VO_2_ at the stage where they were achieved. PFO was determined as the highest fat oxidation achieved in a stage. Fatmax was calculated as a % of VO_2max_, identified by the VO_2_ where the PFO was achieved during the second visit [46].

(1) $$\begin{array}{c} \mathrm{Fat}\;\text{oxidation}\;\left(g/\;\min\;\right)\\ \;=1.695\cdot {\mathrm{VO}}_{2}\;\left(L/\;\min\;\right)–1.701\cdot {\mathrm{VCO}}_{2}\;\left(L/\;\min\;\right) \end{array}$$

#### 2.4.2 Maximal Power and Lactate From MART

The power of the stage was calculated as oxygen cost (mL/kg/min) with Equation (2):
(2)

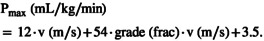

where v = the velocity of the treadmill and grade = the slope of the treadmill expressed as the tangent of the angle with the horizontal [47, 48]. The maximal power (P_max_) was calculated from the oxygen demand of the last completed 20‐s run and from the time of the last run when exhaustion was ensured [47]. Highest lactate from MART was determined as the La_max_.

#### 2.4.3 VO_2max_ and Associated Maximal Speed (sPeak)

VO_2max_ was defined as the highest 30‐s running average from VO_2max_‐tests. Maximal speed from the VO_2max_‐tests (sPeak) was calculated as: last completed stage (km/h) + (duration of unfinished stage [in seconds])/(60 s) · 1 km/h.

#### 2.4.4 Ventilatory Thresholds

VT1 was identified from the incremental tests with the combined method by Gaskill et al. [49]. VT2 was determined from the VO_2max_‐test as VO_2_, when a simultaneous increase in VE/VCO_2_ and Ve/VO_2_ occurred. These VO_2_ values were converted to corresponding speeds (sVT1 and sVT2) by linear fit of speed and VO_2_. The linear fit was based on end‐stage average VO_2_ (60 s in the threshold test, 30 s in the ITs, 20 s in the VO_2max_‐tests), with averaging periods determined by the different lengths of the stages. Thresholds were independently evaluated in a blinded manner by two experienced researchers. If their analyzed VO_2_ values differed by ≤ 2 mL/kg/min, the mean was used; if > 2 mL/kg/min, the evaluators re‐assessed together until consensus was reached. Although the VT1 protocol in visit 1 (3‐min stages, 1 km/h increments) differed from the physiological resilience tests (2‐min stages, 0.8 km/h increments), the intraclass correlation coefficient (two‐way random, absolute agreement) between sVT1 from visit 1 and the first stage of the first physiological resilience test was 0.93 (95% confidence interval: 0.86–0.96), with a coefficient of variation of 2.9%, indicating excellent agreement between the methods without significant difference between the determined thresholds (*p* = 0.140). Results derived from the VO_2max_‐test were not corrected for VO_2_ delay [50], and sVT2 should not be considered to reflect constant speed value. However, sVT2 results were considered to be comparable when using an identical testing and determination protocol.

#### 2.4.5 Running Economy

RE was measured from the six MODs at 85% of sVT1 and expressed as energy cost (kcal/kg/km) which accounts for both oxygen cost and substrate utilization and appears to be more sensitive to measure RE than oxygen cost [51]. Utilization of proteins was assumed negligible. Fat and carbohydrate utilization (g/min) were calculated according to Equations (1) and (3) proposed by Jeukendrup and Wallis [45]:

(3) $$\begin{array}{c} \text{Carbohydrate oxidation}\;\left(g/\;\min\;\right)\\ =\;4.344\cdot {\mathrm{VCO}}_{2}\;\left(L/\;\min\;\right)–3.061\cdot {\mathrm{VO}}_{2}\;\left(L/\;\min\;\right) \end{array}$$

Energy expenditure was calculated by multiplying fat and carbohydrate utilization by 9.75 and 4.07 kcal, respectively. Body mass used in the calculation was the value recorded immediately before each 30‐min MOD where RE was assessed. VO_2_ and VCO_2_ were averaged over the final 2 min of each MOD. The gas analyzer was calibrated before and after each test. If calibration drift exceeded 2%, VO_2_ and VCO_2_ values were corrected linearly.

#### 2.4.6 Heart Rate (HR)

HR was measured during the last 2 min of each MOD. Analysis was done using Kubios HRV Scientific software (version 4.0). For one female recreational runner, the heart rate monitor did not work accordingly and thus in HR analysis 32 subjects were included, of which 17 were recreational and 14 females.

#### 2.4.7 CMJ Height and Maximal Voluntary Contraction (MVC)

CMJ height was calculated from flight time as follows: (9.81 · (flight time)^2^)/8 [52]. MVC was analyzed as the highest force produced during the isometric leg press test using Signal 4.11 software (Cambridge Electronic Design Ltd., Milton, United Kingdom).

#### 2.4.8 Physiological Resilience

Physiological resilience was calculated as percentage change in VO_2max_, sPeak, sVTs, and RE with the following equation: ([POST‐PRE]/PRE) · 100%. For VO_2max_, sPeak and sVT2, the PRE value was the one measured during the second visit and for sVT1 and RE the value was the one measured during the beginning of physiological resilience test. The POST value was the value measured at the end of the physiological resilience test (last IT for sVT1, last MOD for RE and VO2max test for VO2max, sPeak and sVT2). Changes are denoted as Δ%VO_2max_, Δ%sVT2, Δ%sVT1, and Δ%RE.

### 2.5 Statistical Analysis

All results are presented as mean ± standard deviation. Normality of the data was assessed with the Shapiro–Wilk test, histograms and Q‐Q plots and all the data was normally distributed. Difference between non‐fatigued and fatigued VO_2max_, VTs, and RE were tested with paired sample *t*‐tests for the whole sample as well as for competitive and recreational runners, respectively. Association between physiological profile variables and physiological resilience was examined with either Pearson's correlation coefficient or sex‐adjusted partial correlation for variables that may be affected by sex (VO_2max_, sPeak, sVT2, sVT1, P_max_, PFO, and MVC). Correlation coefficients were interpreted as: no or negligible = 0 < *r* < 0.2, low = 0.2 < *r* < 0.4, moderate = 0.4 < *r* < 0.6, high = 0.6 < *r* < 0.8, excellent = 0.8 < *r* < 1.0 Differences between competitive and recreational runners in performance variables and physiological resilience were tested with independent samples *t*‐test. The difference between time‐dependent changes in VT1, RE, and HR between competitive and recreational runners was tested with two‐way repeated ANOVA. Effect sizes were interpreted based on Cohen's *d* for small (0.1–0.3), moderate (0.3–0.5), and large (> 0.5).

## 3 Results

### 3.1 Participant and Trial Characteristics

Participant characteristics are summarized in Table 1 for the participants who completed the whole study. Relative intensity of the four ITs and three MODs was 89.1% ± 0.2% of sVT1 and 90.0% ± 4.1% of VT1 measured during the second visit. Competitive runners ran the four ITs and three MODs during the physiological resilience test with higher average speed than recreational runners (11.8 ± 0.9 vs. 9.3 ± 0.7 km/h, respectively, *p* < 0.001) and they ran longer distance (28.4 ± 2.3 vs. 22.3 ± 1.8 km, respectively, *p* < 0.001). Competitive runners lost body mass on average more than recreational runners during the four ITs and three MODs (−2.2 ± 0.7 vs. −1.6% ± 0.5%, respectively, *p* = 0.003). Of the recruited participants, 12 could not complete the whole study. The reasons for these were injury unrelated to the study (3 competitive runners), sickness in between measurements (5 recreational runners) and scheduling issues (1 competitive and 1 recreational runners). In addition, two of the participants could not complete the physiological resilience test (1 competitive and 1 recreational runner).

**TABLE 1:** Participant characteristics presented as mean (SD).

	Total (*n* = 33)	Competitive (*n* = 15)	Recreational (*n* = 18)	*p* Value (C vs. R)	Males (*n* = 18)	Females (*n* = 15)	*p* Value (M vs. F)
Age (years)	29.8 (6.5)	28.7 (4.5)	30.6 (7.6)	0.422	31.2 (7.2)	28.1 (4.9)	0.181
Height (cm)	174.6 (9.7)	174.1 (9.2)	175.1 (10.1)	0.795	181.7 (6.3)	166.1 (5.3)	< 0.001
Body mass (kg)	70.2 (11.7)	65.3 (9.8)	74.3 (11.5)	0.028	78.9 (7.3)	59.8 (6.3)	< 0.001
Bodyfat (%)	16.1 (4.8)	13.5 (3.4)	18.3 (4.5)	0.002	14.5 (4.7)	18.0 (4.2)	0.032
VO_2max_ (mL/kg/min)	56.2 (7.7)	63.1 (5.6)	50.5 (3.3)	< 0.001	58.2 (7.7)	53.9 (6.9)	0.114
sPeak (km/h)	18.0 (1.6)	19.3 (1.2)	16.9 (1.0)	< 0.001	18.7 (1.6)	17.0 (1.2)	0.002
sVT2 (km/h)	15.3 (1.8)	16.7 (1.4)	14.2 (1.1)	< 0.001	15.9 (1.7)	14.7 (1.6)	0.054
VT2 (%VO_2max_)	90.9 (3.3)	90.9 (3.7)	90.9 (3.0)	0.995	90.5 (4.0)	91.4 (2.3)	0.441
sVT1 (km/h)	12.2 (1.7)	13.8 (1.1)	10.9 (0.9)	< 0.001	12.4 (1.8)	12.0 (1.7)	0.581
VT1 (%VO_2max_)	73.6 (5.3)	75.6 (5.3)	72.0 (5.1)	0.060	71.7 (5.0)	76.0 (5.1)	0.021
RE (kcal/kg/km)	1.00 (0.08)	0.99 (0.06)	1.02 (0.09)	0.273	1.00 (0.08)	1.01 (0.07)	0.775
Fatmax (%VO_2max_)	64.3 (9.5)	69.9 (8.3)	59.6 (7.8)	0.001	61.7 (8.9)	67.4 (9.4)	0.089
PFO (g/min)	0.72 (0.17)	0.80 (0.18)	0.65 (0.15)	0.012	0.81 (0.17)	0.61 (0.11)	< 0.001
P_max_ (mL/kg/min)	93.7 (7.6)	95.0 (8.1)	92.6 (6.9)	0.391	98.9 (4.9)	87.5 (5.2)	< 0.001
La_max_ (mmol/L)	9.1 (3.1)	7.4 (3.1)	10.5 (2.3)	0.003	10.3 (2.8)	7.6 (2.8)	0.014

Abbreviations: C, competitive runners; F, female participants; La max, maximal lactate in maximal anaerobic running test; M, male participants; PFO, peak fat oxidation; P_max_, maximal power in maximal anaerobic running test; R, recreational runners; RE, running economy; sPeak, maximal speed in VO_2max_‐test; sVT1, speed associated with ventilatory threshold 1; sVT2, speed associated with ventilatory threshold 2; VO_2max_, maximal oxygen uptake.

Competitive and recreational runners differed in VO_2max_, sPeak, sVT2, sVT1, fatmax, PFO, and La_max_ in MART but not in P_max_ or RE in non‐fatigued state (Table 1). There was no difference between competitive and recreational runners in CMJ (*p* = 0.15) or MVC (*p* = 0.05).

### 3.2 Changes in Physiological Profile During Physiological Resilience Test

When comparing non‐fatigued and fatigued physiological profiles among all subjects, a significant change in every variable was detected during the physiological resilience test (Table 2). Competitive runners showed changes in all variables (*p* < 0.02), except MVC (*p* = 0.38). Recreational runners showed changes in all variables (*p* < 0.01) except VO_2max_ (*p* = 0.06) and CMJ (*p* = 0.37).

**TABLE 2:** Mean ± SD results of the performance and physiological variables between the PRE and POST physiological resilience tests and the percent change in them (∆%).

Variable	PRE	POST	∆%	*p*	Effect size
VO_2max_ (mL/kg/min)	56.2 ± 7.8	54.9 ± 7.5	−2.3 ± 4.1	0.002	−0.58
sPeak (km/h)	18.0 ± 1.7	16.8 ± 1.7	−6.4 ± 3.7	< 0.001	−1.69
sVT2 (km/h)	15.3 ± 1.8	14.5 ± 1.6	−5.5 ± 5.6	< 0.001	−0.98
sVT1 (km/h)	12.1 ± 1.5	11.6 ± 1.7	−4.4 ± 4.8	< 0.001	−0.92
RE (kcal/kg/km)	1.00 ± 0.08	1.05 ± 0.07	5.0 ± 3.7	< 0.001	1.30
CMJ (cm)	31.9 ± 7.4	33.2 ± 7.4	4.5 ± 8.5	0.004	0.49
MVC (N)	3291 ± 1123	3099 ± 989	−4.6 ± 11.0	0.005	−0.48

Abbreviations: CMJ, countermovement jump; MVC, maximal voluntary contraction in isometric leg press; RE, running economy; sPeak, maximal speed in VO_2max_‐test; sVT1, speed associated with ventilatory threshold 1; sVT2, speed associated with ventilatory threshold 2; VO_2max_, maximal oxygen uptake.

### 3.3 Differences Between Competitive and Recreational Runners in Physiological Resilience

**FIGURE 3:**
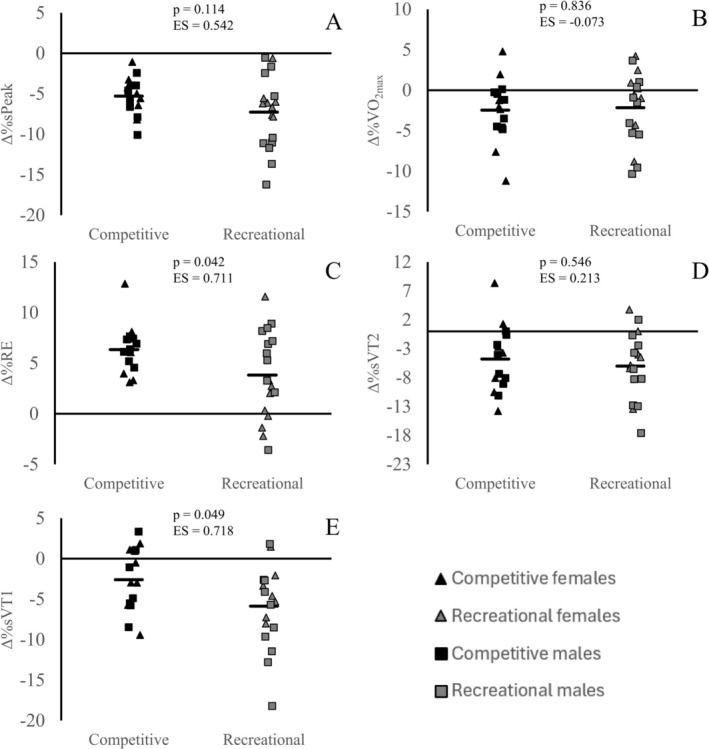
Individual changes (∆% = 100 · (POST‐value—PRE‐value)/PRE‐value) in the performance variables of the competitive and recreational runners. Males are displayed as squares and females as triangles. sPeak, maximal speed in V̇O_2max_‐test; sVT1, speed associated with ventilatory threshold 1; sVT2, speed associated with ventilatory threshold 2; RE, running economy; V̇O_2max_, maximal oxygen uptake. Black horizontal lines show group averages. *p* Values and effect sizes (ES) correspond to comparisons between competitive and recreational runners in distinct variables.

**FIGURE 4:**
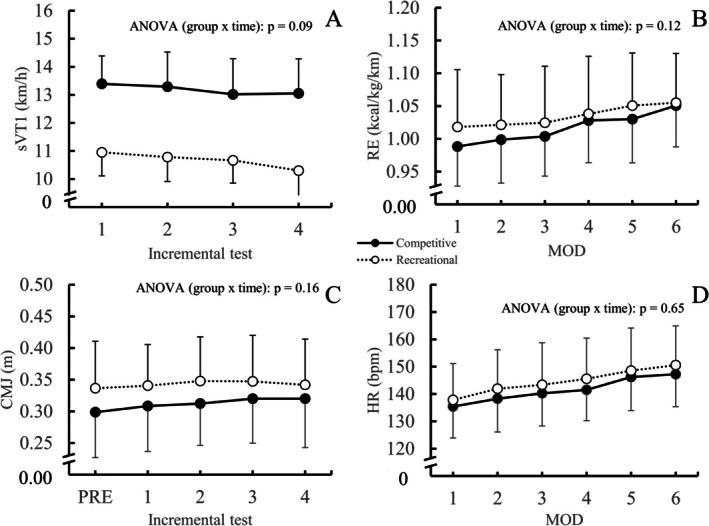
Time‐dependent changes in (A) speed at ventilatory threshold 1 (sVT1), (B) running economy (RE), (C) countermovement jump (CMJ), and (D) heart rate (HR) in competitive (black circles) and recreational runners (white circles) in the physiological resilience test.

Differences between competitive and recreational runners in physiological resilience were observed only in Δ%sVT1 (−2.6% ± 3.9% vs. −5.9 ± 5.0, respectively) and Δ%RE (6.3% ± 2.4% vs. 3.8 ± 4.3, respectively). No differences were detected for Δ%VO_2max_ (−2.5 ± 3.9 vs. −2.2 ± 4.4), Δ%sPeak (−5.3 ± 2.3 vs. −7.3 ± 4.4), or Δ%sVT2 (−4.8 ± 5.7 vs. –6.0 ± 5.6) (Figure 3). ANOVA analysis showed no group × time interaction between competitive and recreational runners in sVT1, RE, CMJ, or HR during the physiological resilience test (Figure 4).

### 3.4 Factors Associated With Physiological Resilience

**FIGURE 5:**
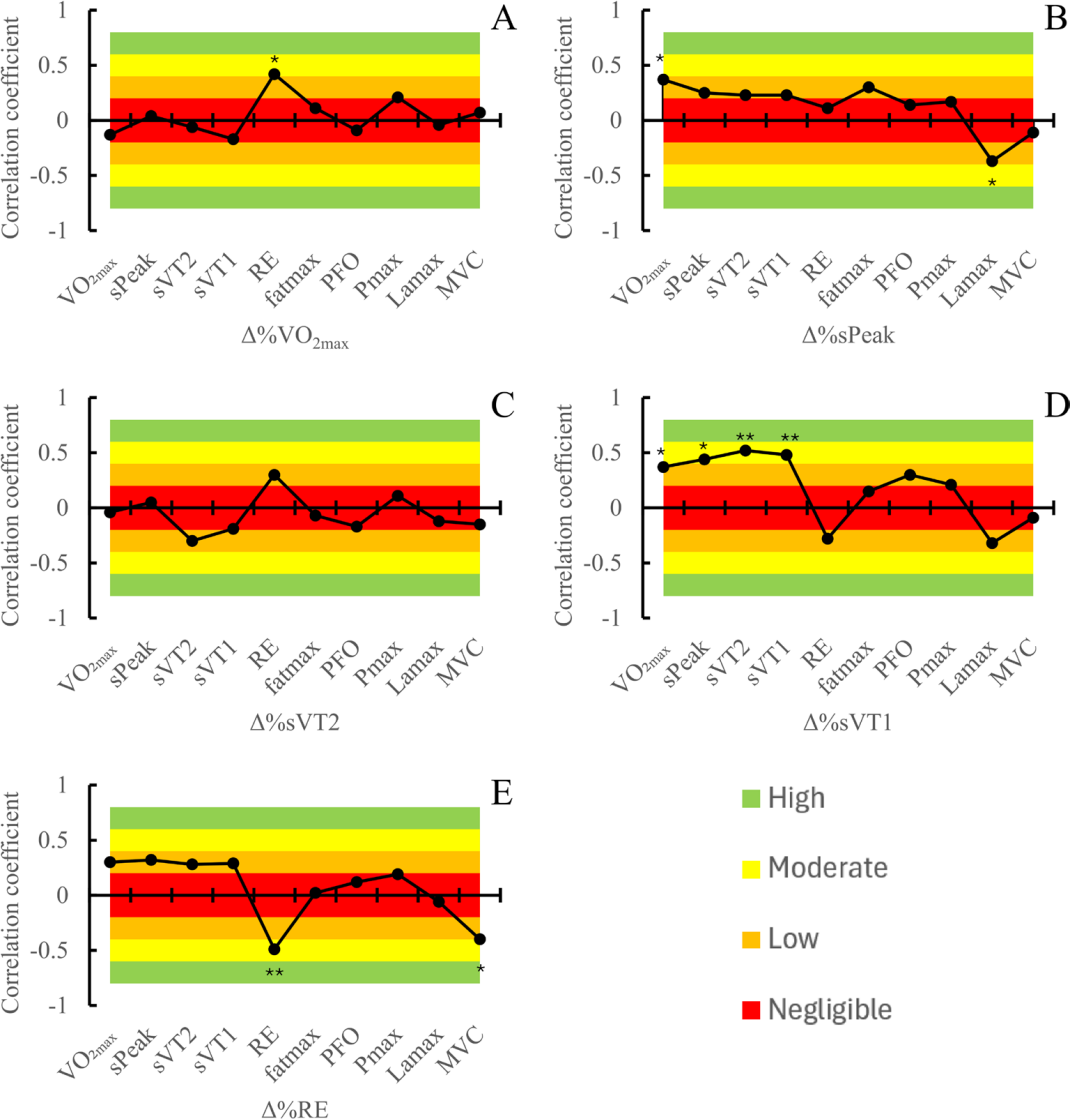
Correlations coefficients with changes in physiological profile variables and determining factors of endurance performance. Sex‐adjusted partial correlation was used for variables that may be affected by sex (VO_2max_, sPeak, sVT2, sVT1, PFO, P_max_, and MVC). In other variables Pearson's correlation coefficient was used. Color codes are the following: Red = negligible correlation, orange = low correlation, yellow = moderate correlation, green = high correlation.

Correlation analysis demonstrates that smaller Δ%sPeak was associated with higher VO_2max_ (*r* = 0.37, *p* = 0.04) and lower La_max_ (*r* = −0.37, *p* = 0.03). Smaller Δ%sVT1 was positively associated with higher VO_2max_ (*r* = 0.37, *p* = 0.04), sVT2 (*r* = 0.52, *p* = 0.002), sVT1 (*r* = 0.48, *p* = 0.006), and sPeak (*r* = 0.44, *p* = 0.01), while smaller change in Δ%RE was associated with worse RE (*r* = −0.49, *p* = 0.004) and higher MVC (*r* = −0.40, *p* = 0.02). Smaller Δ%VO_2max_ was associated with worse RE (*r* = 0.42, *p* = 0.02). All correlations between changes in physiological profile and variables determining endurance performance are presented in Figure 5.

## 4 Discussion

The main findings of the study were (1) that higher aerobic fitness (VO_2max_, sPeak, sVT2, and sVT1) and higher maximal strength of the leg extensors and anaerobic capacity (MVC and La_max_) were associated with better physiological resilience of distinct variables during the physiological resilience test, and (2) recreational runners had a higher decline in Δ%sVT1 and lower in Δ%RE than competitive runners but did not differ in other physiological resilience variables. Based on these findings, it appears that there are only small differences between competitive and recreational runners and that physiological resilience is comprised of multiple different physiological systems and the ability to resist the decline in them is determined by the respective systems.

### 4.1 Deterioration of the Physiological Profile

On average, the physiological profile deteriorated. Based on effect sizes, the largest declines were observed in sPeak (ES = −1.69) and RE (ES = 1.30). These variables were also the most reliable metrics of physiological resilience in the previously reported reliability analysis of the current dataset [41]. In addition, large declines were observed in VO_2max_, sVT2, and sVT1 (ES = −0.98 to −0.58). The deterioration of the variables was similar as reported in previous studies for all variables except VO_2max_ [7, 8, 10]. In our study, VO_2max_ declined −2.5 ± 3.9 and −2.2% ± 4.4%, respectively, for competitive and recreational runners. In contrast, Zanini et al. [10] showed a larger decrement of VO_2max_ after 90 and 120 min of heavy intensity running (−3% and −7%) and a similarly larger decline of VO_2max_ after half‐marathon has been reported (−6.5%) [53]. The difference between our findings and previous studies might be related to the difference in the protocol of the fatiguing task and incremental test to determine VO_2max_.

### 4.2 Associations Between Determining Factors of Endurance Performance and Physiological Resilience

To the best of our knowledge, this is the first study to investigate associations between multiple endurance performance determining factors and deterioration of the variables in the Joyner model during endurance running. Previous studies have reported contradictory results related to factors associated with physiological resilience. Spragg et al. [18] reported that the change of critical power was associated with all performance variables they measured, while the studies by Clark et al. [3, 12, 33, 54] showed no correlation with change in critical power and any baseline values of the variables thought to explain endurance performance. Similar results were obtained in the studies by Gallo et al. [16] and Stevenson et al. [17]. Findings of the current study settle in between these extremities, finding distinct associations for different resilience variables. Based on the results of our study, it appears that both strength and aerobic fitness are important for physiological resilience. One important finding was that there was no single variable associated with declines in all the variables. The variables in the Joyner model are related to different physiological systems. For example, VO_2max_ is related mainly to lungs' oxygen diffusion capacity, cardiac output, blood oxygen carrying capacity, and muscles' oxygen extraction capacity [13], while VTs are related to the muscles' oxidative capacity [15], and RE is related to both metabolic and mechanical efficiency of the muscle‐tendon system [14]. Speeds at VO_2max_ and ventilatory thresholds are related to the determinants of VO_2max_ and VTs, but also RE [55]. As physiological resilience is defined as a decline in these variables during prolonged exercise, the decline indicates that the determining factors of the said variable are affected during the exercise. As the variables are determined by different physiological systems, it is logical that there is not one variable associated with the declines in all the variables. Therefore, training programs aiming to improve physiological resilience should be targeted to the specific resilience variables. Future studies should investigate which one of the physiological resilience variables is the most relevant for race performance, as this would improve the training prescription further.

Higher aerobic fitness was related to better resilience of sPeak and sVT1. As only VO_2max_ was related to ∆%sPeak, it may be that better ∆%sPeak requires higher overall capacity of the oxygen transport chain [56]. In contrast, ∆%sVT1 was also related to both VTs and sPeak. VTs are related to oxidative capacity of the muscles [15]. This finding is contradictory to previous studies that did not find significant correlations between decline in sVT1 and determining factors of endurance performance during cycling [16, 17]. The deterioration of the ventilatory thresholds is caused by either decrease in the metabolic power at the threshold or decrease in efficiency, with loss of metabolic power appearing to be the main cause during endurance running [7]. It may be that better overall aerobic fitness, and associated with higher oxidative capacity, capillary density, and mitochondrial density, improves the ability to sustain homeostasis and thus improves the ability to resist the decline in metabolic power and subsequently sVT1. It was surprising that no variable was associated with Δ%sVT2, although sVT2 declined. The reason for this might be that the exercise was conducted at moderate intensity which might be more challenging for sVT1 than sVT2. VT2 reveals the maximal metabolic state where homeostasis can be sustained, and it is related to the ability to resist acidosis from occurring. The lack of associations might arise also from the determination method of VT2 previously described in the methods section.

Based on the results of the current study, it appears that maximal strength of the leg extensors may play a part in preserving RE during prolonged moderate‐intensity running. A previous study found that strength training improved the ability to sustain RE during prolonged running [20] and similar results have been obtained from cycling [21, 22] and cross‐country skiing [23]. One cause for this might be that higher maximal strength lowers the relative force needed during prolonged running, and therefore the need to recruit higher level motoneurons [24]. Higher threshold motoneurons and the muscle fibers they innervate are less efficient [29], so therefore the limitation of their recruitment might limit the deterioration of RE. Furthermore, higher maximal strength has been associated with tendons' greater ability to store elastic energy and thus improve RE [57]. In addition, during prolonged running, the stiffness of the tendons deteriorates, resulting in impaired RE [58]. Thus, higher maximal strength may also buffer the deterioration of the elastic properties of the tendons. Future studies should investigate this in more detail.

Higher proportion of type II fibers has been associated with faster fatigability during submaximal exercise [25, 28], while higher proportion of type I fibers has been proposed to be associated with better resilience [5]. La_max_ has been associated with the percentage of type II muscle fibers of the individual [26]. The present results indicate that higher La_max_, and thus possibly higher proportion of type II fibers, is detrimental for the ability to sustain maximal aerobic performance (sPeak) during prolonged moderate‐intensity running. The possible effect of the fiber type distribution on this association is backed up by the fact that P_max_ was not related to the decline in sPeak. Blood lactate concentration is determined by the lactate production from glycolytic fibers and lactate uptake of the oxidative fibers [59]. Therefore, higher La_max_ indicates higher glycolytic capacity in relation to oxidative capacity. This indicates that regardless of the maximal anaerobic power achieved in MART, the end lactate was related to ∆%sPeak.

Differences between the current study and previous studies probably arise from the different exercise modalities, as the muscle work is different between running and cycling; running includes both concentric and eccentric muscle work, while cycling is primarily concentric muscle work [60]. Also, different exercise intensities may affect the determinants of physiological resilience, and previous studies have used either severe [18] or moderate intensity exercise [16, 17]. Different intensity domains have distinct acute physiological responses and routes to fatigue [30], thus possibly affecting the determinants of resilience. Another challenge is the absence of a standardized approach for assessing physiological resilience. Moreover, resilience metrics have been shown to vary considerably in their reliability and variability, making it more difficult to identify the determinants of metrics that exhibit greater variation [41]. Added to the variability, studies have had rather small sample sizes (10–14 participants) and thus some variables may have shown up as significant or non‐significant by statistical chance.

### 4.3 Differences Between Competitive and Recreational Runners in Physiological Resilience

It is currently unclear how different level runners differ in physiological resilience or if there are any differences. Comparing runners of different fitness levels would tell if a certain variable of physiological resilience would be sensitive to differentiating runners and thus possibly allow to follow the improvement in athletes. Competitive and recreational runners differed in every performance variable at baseline, except P_max_ and RE. Recreational runners showed larger deterioration in sVT1 than competitive runners, but in contrast competitive runners exhibited larger deterioration in RE. In addition, the effect size for Δ%sPeak was large between competitive and recreational runners, although the difference was not significant. These findings propose that sVT1, and possibly sPeak, may allow to differentiate runners of different fitness levels when the test is done across standardized time.

Δ%RE was similar as previously reported [11]. It was surprising that competitive runners had greater decrement in economy than recreational runners, as previous studies have reported opposite results [9, 11]. The reason for this might be that the exercise was performed with standardized time, so competitive runners ran longer distances compared to recreational runners and thus performed more work. This might also explain why other resilience variables did not differ between the groups. Mateo‐March et al. [61] showed that high‐caliber cyclists exhibit lower decline in maximal mean power outputs compared to low‐caliber cyclists after a certain amount of work. Additionally, as RE was expressed relative to body mass, and body mass changed during the protocol, the amount of lost body mass affects changes in RE. Competitive runners lost more body mass on average than recreational runners, and therefore RE was affected more in the competitive runners than in the recreational runners. Post hoc analysis showed that the increase in absolute energy expenditure did not differ between the groups highlighting the effect of body mass on the results.

### 4.4 Limitations

This was a cross‐sectional study and thus the results do not show causality. Future studies should investigate whether improving, for example, strength or aerobic fitness transfers to improved physiological resilience, and if either is better than the other. The physiological resilience test was performed over time and not over distance. This causes differences in the energy expenditure between the groups as well as between individuals. There is evidence that performance declines more after larger energy expenditures [61]. This might have caused the non‐significant differences between competitive and recreational runners. The level of the athletes was determined as Tier 2 for recreational runners and Tier 3 or higher for competitive runners [37]. Although the groups differed in most of the performance variables, larger differences in training status for the groups may have given better picture of the effect of different fitness levels on physiological resilience. The physiological resilience test was conducted in moderate intensity domain, which is not the typical intensity where endurance running races are performed. For example, previous studies have reported larger decoupling in RE after a marathon [62] and larger decrement in VO_2max_ after a half marathon [53] or heavy intensity running [10] than in the current study. No studies to date have investigated the difference in physiological resilience between different intensity domains, so the irreversibility of the results to other intensities remains unclear. Thresholds can be assessed with multiple different methods and thus different methods to determine the ventilatory thresholds in the current study could have changed the results. This is also evident for the different tests to assess VT1 and VT2 in the current study, and this might have affected the present results. Fatmax testing was conducted while walking, which may have affected the results compared to a situation where the test had been conducted while running.

## 5 Conclusions

This study revealed that the ability to sustain sVT1 during prolonged running at moderate intensity was associated with multiple variables resembling aerobic fitness, while the ability to sustain RE with maximal strength of the leg extensors. Higher VO_2max_ and lower anaerobic capacity (and thus possibly muscle fiber type distribution) were related to better ability to sustain maximal aerobic performance (sPeak). Additionally, recreational runners had a larger decline in sVT1, and in sPeak based on effect size, and lower decline in RE than competitive runners but not in other physiological resilience variables during prolonged moderate intensity running. Physiological resilience, therefore, appears to be associated with multiple different physiological systems. Interventions or training programs should consequently target specific modalities to improve selected resilience variables that are of interest.

## 6 Perspective

These findings represent evidence that physiological resilience, defined as the ability to resist decline in physiological profile, is related to endurance performance, is determined by multiple different physiological systems. Also, recreational runners had worse resilience of sVT1, while better resilience of RE. It appears that resilience of RE may be associated with greater strength of the leg extensors, while resilience of sVT1 and maximal speed in an incremental test is more related to aerobic fitness, and possibly fiber type distribution. In the future, more mechanistic and interventional studies are needed to investigate more extensively the determinants of physiological resilience and how it might be trained. For example, the causality of the current findings needs to be investigated by interventional studies targeting to improve maximal strength of leg extensors to improve ability to sustain RE or similarly to improve aerobic fitness to improve resilience of sVT1 or sPeak. In the current study, physiological resilience was measured as deterioration in five different variables (VO_2max_, sPeak, VTs, and RE). Future studies should investigate which one of these variables is the most relevant for race performance across different distances. This information would help to improve training prescription for athletes.

## Funding

The study received a grant from the Finnish Sports Research Foundation (Suomen urheilututkimussäätiö).

## Ethics Statement

The study was approved by the ethics committee of the University of Jyväskylä (1581/13.00.04.00/2024).

## Conflicts of Interest

The authors declare no conflicts of interest.

## Data Availability

The data that support the findings of this study are available from the corresponding author upon reasonable request.
